# Dental health behavior of parents of children using non-fluoride toothpaste: a cross-sectional study

**DOI:** 10.1186/1472-6831-13-74

**Published:** 2013-12-29

**Authors:** Junko Ota, Tatsuo Yamamoto, Yuichi Ando, Jun Aida, Yukio Hirata, Seishiro Arai

**Affiliations:** 1 Department of Dental Sociology, Kanagawa Dental University Graduate School of Dentistry, 82 Inaoka-cho, Yokosuka, 238-8580 Kanagawa, Japan; 2 Department of Health Promotion, National Institute of Public Health, Wako, Saitama, Japan; 3 Department of International and Community Oral Health, Tohoku University Graduate School of Dentistry, Sendai, Miyagi, Japan; 4 8020 Promotion Foundation, Chiyoda-ku, Tokyo, Japan

**Keywords:** Dental health behavior, Parents, Elementary school children, Non-fluoride toothpaste

## Abstract

**Background:**

One of the dental health goals of Health Japan 21, in which the Japanese government clarified its health policy, was to ensure the use of fluoride toothpaste in 90% or more of schoolchildren. This goal was not achieved. The aim of this cross-sectional questionnaire study was to evaluate the characteristics of parents whose children use non-fluoride toothpaste.

**Methods:**

In December 2010, questionnaire forms were sent to 18 elementary schools or school dentists. Students (6-12 years old) were asked to take the forms home for their parents to fill in, and to bring the completed questionnaire to school. The collected questionnaires were mailed from schools to the author’s institution by the end of March 2011. The relationship between fluoride in toothpaste and reasons for choice of toothpaste, the child’s toothbrushing habits, and attitude toward child caries prevention was examined in the 6,069 respondents who answered all the questions for the analyses and indicated that their children use toothpaste.

**Results:**

Non-fluoride toothpaste users accounted for 5.1% of all toothpaste users. Among the children using non-fluoride toothpaste, significantly greater numbers gave ‘anti-gingivitis’, ‘halitosis prevention’ or ‘tartar control’ as reasons for choice of toothpaste; did not give ‘has fluoride’, ‘is cheaper’ or ‘tastes good’ as reasons for choice of toothpaste; or used toothpaste sometimes, or were in 4th - 6th grades. There was no significant relationship between use of non-fluoride toothpaste and measures taken for caries prevention in children. Multilevel (first level: individual, second level: school) logistic regression analysis indicated that use of non-fluoride toothpaste was significantly related to: giving ‘anti-gingivitis’ (odds ratio: 1.44) as a reason for choice of toothpaste; not giving ‘has fluoride’ (0.40), ‘tastes good’ (0.49) or ‘is cheaper’ (0.50) as the reason for choice of toothpaste; to toothbrushing less often (twice a day: 1.34, once a day or less: 1.46) and to using toothpaste less often (sometimes: 1.39).

**Conclusions:**

It is necessary to teach parents that dental caries is the dental health issue with the highest priority for children, and therefore fluoride toothpaste should be used.

## Background

The incidence of dental caries has decreased in many countries over the last few decades, and many experts have attributed this mainly to the widespread use of fluoride toothpaste [1]. Water is not fluoridated in Japan, and the use of fluoride toothpaste serves as a public health measure for the prevention of caries. However, the spread of fluoride toothpaste has been slower in Japan than in other countries [2].

In 2000, the Ministry of Health and Welfare (now the Ministry of Health, Labour and Welfare) put forward the ‘Health Japan 21’ plan, which set specific health targets for 2010. Regarding dental health, the plan endorsed the use of fluoride toothpaste, and aimed for its use in 90% or more of schoolchildren [3]. However, the final assessment of Health Japan 21 indicated that although there had been improvement in the figures, the numerical target was not achieved (percentage of schoolchildren using fluoride toothpaste: 86.3) [4]. The use of fluoride toothpaste has thus not reached a satisfactory level.

In view of future efforts to increase the proportion of children using fluoride toothpaste, understanding the characteristics of parents whose children do not use fluoride toothpaste may provide useful. Children who do not use fluoride toothpaste can be divided into two groups: those not using toothpaste at all and those using non-fluoride toothpaste. Our previous study showed that the most frequent reason (multiple answers) for not using toothpaste was bad taste (40%), which was followed by difficulty in brushing due to foaming (20%), advice of dentist or dental hygienist (14%), abraded teeth (6%), and belief that it has no effect on oral health (6%) [5]. This study aims to investigate the characteristics of parents whose children use non-fluoride toothpaste, specifically examining the reasons for toothpaste choice, tooth brushing habits of children, and attitude toward child caries prevention.

## Methods

### Participants

In this cross-sectional questionnaire study, elementary schools (age of children ranged from 6 to 12 years old) were selected by first selecting school dentists from the register of organizations such as the Japan Dental Association and the Japanese Society for Oral Health as expedient. Japan was divided into six regions: Hokkaido-Tohoku, Kanto, Chubu, Kinki, Chugoku-Shikoku and Kyushu-Okinawa, and at least five school dentists were selected from each region, so as to minimize bias across the country. However, because the school dentists must explain the details of the survey to the schools, we selected dentists who agreed to collaborate, such as those we knew or those who were introduced to us by mutual acquaintances. As a result, 18 elementary schools (17 public schools and one private school) located in the six regions (Hokkaido-Tohoku (one school in one city), Kanto (five schools in four cities), Chubu (six schools in three cities, one town and one village), Kinki (one school in one city), Chugoku-Shikoku (one school in one city and one in one town) and Kyushu-Okinawa (three schools in one city and one town)) consented to participate in the survey.

**Figure 1:**
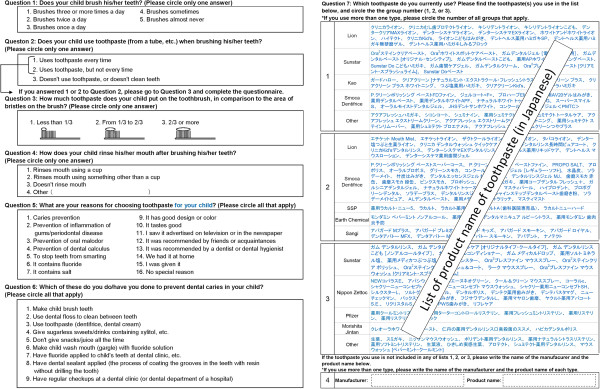
Excerpt from the questionnaire form (only questions used in this study are included).

Questionnaires were distributed to the parents of all 8,490 children at the participating schools, and 6,931 responded (response rate: 81.6%). The following questionnaires were excluded: 34 in which the sex or grade level of the child was not mentioned, 348 in which the child did not use toothpaste or did not brush his/her teeth (Question 2, response 3 (Figure 1)), 41 in which it was unclear whether or not the child used toothpaste (no response to Question 2), 79 in which the toothpaste used was not known (no response to Question 7 “Which toothpaste do you currently use? Please find the toothpaste(s) you use in the list below, and circle the group number (1, 2, or 3)”. (Figure 1)), and 360 in which there was no response to one or more of the analyzed items (Questions 1, 3–6). The data of the remaining 6,069 children (2,921 boys, 3,148 girls) were subjected to analysis (Table 1).

**Table 1:** Gender and grade of subjects

**Grade (age in years)**	**Boys**	**Girls**	**Total**
1 (6-7)	496	508	1,004
2 (7-8)	480	536	1,016
3 (8-9)	495	569	1,064
4 (9-10)	483	526	1,009
5 (10-11)	487	527	1,014
6 (11-12)	480	482	962
Total (6-12)	2,921	3,148	6,069

Total population and area of each municipality (city, town or village) were obtained from the national survey data, and population density was calculated for each municipality. Total taxable income and number of taxable people were also obtained from the national survey data, and mean income per population was calculated for each municipality.

### Survey method

The questionnaires for every child in each participating school were sent together in December 2010 either to the school or to the school dentist. The children were given the forms at school, and were asked to take the forms home and have their parents or guardian fill them in. The questionnaire was a partially modified version of the questionnaire used by Hirata et al. [6] to which some additions had been made [7] (Figure 1). The children were asked to bring the completed questionnaire to school. Then the questionnaires were collected and sent from each school to the author’s institution by mail by the end of March 2011.

### Analysis

The participants were divided into two groups: people using fluoride toothpaste (Question 7 “Which toothpaste do you currently use? Please find the toothpaste(s) you use in the list below, and circle the group number (1, 2, or 3)”. (Figure 1) response 1, and those who indicated a fluoride toothpaste in Question 7 response 4) and people using non-fluoride toothpaste (Question 7 response 2 or 3, and those who indicated a non-fluoride toothpaste in Question 7 response 4). First, univariate odds ratios (ORs) for sex, grade level, reason for toothpaste selection (Question 5), toothbrushing method (Question 1, frequency of toothbrushing; Question 2, frequency of toothpaste use; Question 3, amount of toothpaste used; Question 4, rinsing), and behavior to prevent caries (Question 6) were calculated. The dependent variable in the model is 1 if the child was a non-fluoride toothpaste user and 0 if the child was a fluoride toothpaste user. Questions with less than 10% of yes or no answers (reason for toothpaste selection (Question 5) and behavior to prevent caries (Question 6)) were excluded from the analyses. Because socioeconomic condition is associated with oral health behavior [8], association of non-fluoride use with type of school (private schools are generally more expensive than public schools and the type of school may reflect the socioeconomic status of parents), and population density and mean income per population in each municipality was analyzed.

Then, a multilevel logistic regression model with random intercepts and fixed slopes was used to calculate multilevel ORs, taking ‘school’ into account (second level), after adding for all independent variables, each of which showed association with the use of non-fluoride toothpaste at the significance level of *p* < 0.15. Before adding the independent variables, correlation of variables was checked using Spearman’s correlation coefficient. All statistical analyses were conducted using IBM SPSS Statistics version 20 (IBM Co., Armonk, NY, USA) and MLwiN 2.28 software package (Centre for Multilevel Modelling, University of Bristol, Bristol, UK).

### Ethical considerations

This study was conducted in full accordance with ethical principles, including the World Medical Association Declaration of Helsinki. It was ensured that the questionnaire form was anonymous, with no way of identifying any individual. This study was approved by the Ethical Committee of Kanagawa Dental University (approval no. 134).

## Results

Non-fluoride toothpaste users accounted for 5.1% of all toothpaste users. Among the participants using non-fluoride toothpaste, significantly greater numbers gave ‘anti-gingivitis’, ‘halitosis prevention’ or ‘tartar control’ as reasons for choice of toothpaste; did not give ‘has fluoride’, ‘is cheaper’ or ‘tastes good’ as reasons for choice of toothpaste; or used toothpaste sometimes, or were in 4th-6th grade (*p* < 0.05) (Table 2). No significant relationship was found between the presence or absence of fluoride in the toothpaste being used and behavior for preventing caries in the children (*p* > 0.05).

**Table 2:** Univariate relationship between toothpaste fluoride content and individual-, school- and municipality (city, town or village)-level characteristics

		**n**	**% of non-fluoride toothpaste users**	Univariate odds ratio^*^	*p*-value^*^
Gender	Boys	2,921	4.9	1.00	
	Girls	3,148	5.3	1.09	0.469
Grade	1-3	3,084	4.5	1.00	
	4-6	2,985	5.7	1.27	0.041
Reason for choice of toothpaste					
Caries prevention	No	2,071	5.6	1.00	
	Yes	3,998	4.8	0.85	0.167
Periodontal disease prevention	No	5,029	4.6	1.00	
	Yes	1,040	7.6	1.71	<0.001
Measure against oral malodor	No	4,805	4.8	1.00	
	Yes	1,264	6.4	1.37	0.019
Dental calculus prevention	No	5,435	4.9	1.00	
	Yes	634	7.1	1.49	0.017
Contains fluoride	No	3,201	7.1	1.00	
	Yes	2,868	2.9	0.40	<0.001
Inexpensive	No	4,844	5.7	1.00	
	Yes	1,225	2.8	0.47	<0.001
Good taste	No	4,499	6.0	1.00	
	Yes	1,570	2.6	0.47	<0.001
Toothbrushing habits of children					
Frequency of toothbrushing	3 or more times a day	1,754	4.3	1.00	
	Twice a day	3,281	5.3	1.25	0.109
	Once a day or less	1,034	5.9	1.40	0.055
Frequency of toothpaste use	Every time	4,196	4.7	1.00	
	Sometimes	1,873	6.0	1.30	0.029
Amount of toothpaste used	2/3 or greater	755	4.4	1.00	
(measured against bristles)	1/3 to less than 2/3	3,567	5.2	1.20	0.351
	Less than 1/3	1,747	5.3	1.22	0.346
Rinsing	Cup used	4,971	4.9	1.00	
	Other than cup	1,060	6.1	1.28	0.090
	Doesn’t rinse/other	38	7.9	1.67	0.394
Attitude toward child caries prevention					
Makes child use dental floss	No	5,076	5.0	1.00	
	Yes	993	5.5	1.11	0.501
Makes child use toothpaste	No	2,441	5.6	1.00	
	Yes	3,628	4.8	0.85	0.181
Don’t give snacks/juice all	No	4,232	5.2	1.00	
the time	Yes	1,837	4.9	0.94	0.628
Makes child wash mouth with	No	4,945	5.2	1.00	
fluoride solution	Yes	1,124	4.9	0.95	0.717
Have fluoride applied to surface	No	3,768	5.3	1.00	
of child’s teeth	Yes	2,301	4.8	0.90	0.367
Have sealant applied to child’s	No	5,317	5.2	1.00	
teeth	Yes	752	4.4	0.84	0.338
Regular dental checkups	No	3,909	5.1	1.00	
for child	Yes	2,160	5.1	1.01	0.935
Public or private school	Public	5,759	5.2	1.00	
	Private	310	6.8	1.33	0.226
Population density of city, town or	Rural-agricultural (< 1,000)	1,608	5.2	1.00	
village (/km^2^)	Urban (1,000-3,999)	1,471	5.2	1.05	0.721
	Metropolitan (≥ 4,000)	2,990	5.0	1.05	0.718
Mean income per population of city	20,000-24,999	577	4.2	1.00	
town or village (US$)	25,000-29,999	2,053	5.0	1.22	0.398
	30,000-34,999	3,119	5.2	1.26	0.298
	35,000-39,999	320	6.6	1.62	0.117

^*^ Dependent variable: non-fluoride toothpaste user (1), fluoride toothpaste user (0).

The results of the multilevel logistic regression analysis with use of non-fluoride (1) and fluoride (0) toothpaste as dependent variables are shown in Table 3. Intercept only model showed non-significant school-level variance (0.004, standard error = 0.020). Odds ratios (95% confidence intervals) were significantly higher when responding that frequency of toothbrushing was twice a day (1.34, 1.00-1.78) and once a day or less (1.46, 1.02-2.09) and that toothpaste was used sometimes (1.39, 1.08-1.78) and the reason for toothpaste choice was ‘anti-gingivitis’ (1.44, 1.07-1.95); and for not responding the reason was ‘has fluoride’ (0.40, 0.31-0.52), ‘tastes good’ (0.49, 0.35-0.68), and ‘is cheaper’ (0.50, 0.35-0.72). Spearman’s correlation coefficient of all independent variables ranged from -0.191 to 0.342.

**Table 3:** Results of multilevel logistic regression analyses

		**Odds ratio**	**95% confidence interval**	***p*-value**
Fixed effect				
Individual-level variables				
Grade	1-3	1.00		
	4-6	1.09	(0.86-1.38)	0.481
Reason for choice of toothpaste				
Periodontal disease prevention	No	1.00		
	Yes	1.44	(1.07-1.95)	0.017
Measure against oral malodor	No	1.00		
	Yes	1.20	(0.89-1.61)	0.231
Dental calculus prevention	No	1.00		
	Yes	1.31	(0.90-1.90)	0.154
Contains fluoride	No	1.00		
	Yes	0.40	(0.31-0.52)	<0.001
Inexpensive	No	1.00		
	Yes	0.50	(0.35-0.72)	<0.001
Good taste	No	1.00		
	Yes	0.49	(0.35-0.68)	<0.001
Toothbrushing habit of children				
Frequency of toothbrushing	3 or more times a day	1.00		
	Twice a day	1.34	(1.00-1.78)	0.047
	Once a day or less	1.46	(1.02-2.09)	0.037
Frequency of toothpaste use	Every time	1.00		
	Sometimes	1.39	(1.08-1.78)	0.009
Rinsing	Cup used	1.00		
	Other than cup	1.21	(0.90-1.61)	0.204
	Doesn’t rinse/other	2.26	(0.67-7.64)	0.188
Constant		0.05	(0.04-0.08)	<0.001
Random effects				
School-level variance (standard error)		0.000	0.000	

Dependent variable: non-fluoride toothpaste user (1), fluoride toothpaste user (0).

Intercept only model showed non-significant school-level variance (0.004, standard error = 0.020).

## Discussion

The results in the present study show that choosing toothpaste for ‘anti-gingivitis’ was significantly related to use of non-fluoride toothpaste. Bearing in mind the current prevalence of periodontal disease among elementary school children (in the 2011 Survey of Dental Diseases [9], approximately 25% of 5-9 year-olds had bleeding on probing in the Community Periodontal Index, which is a relatively low detection rate for periodontal disease in school children, lower than the prevalence of dental caries (including experience) in deciduous and permanent teeth of 5-9 year-olds (66.4%)), it seems unlikely that parents purchase anti-gingivitis toothpaste for their children. Rather, it is possible that the parents of children using non-fluoride toothpaste are conscious of their own gingivitis when purchasing toothpaste, and subsequently share this toothpaste with their children.

In addition, among children who use non-fluoride toothpaste, significantly more parents responded that toothpaste was used sometimes rather than every day. Moreover, children with lower frequency of toothbrushing were more likely to use non-fluoride toothpaste. In a prior study, bad taste was the most common reason (approx. 40%) for children not using toothpaste [5]. Taste was a major factor in selecting toothpaste according to mothers of preschool children in Malaysia [10] and of teenagers in Sweden [11]. Generally speaking, anti-gingivitis toothpastes often have strong tastes. Children usually use the toothpaste available at home [11]. Children sharing their parents’ anti-gingivitis toothpaste probably use toothpaste with less frequency because they find adult flavors such as mint disagreeable.

A previous study has shown that toothbrushing behavior of children was significantly associated with that of parents [12]. Also, a survey on toothpaste sharing between parents and children reported that 33% of children in the lower grades of elementary school and 53% of children in the upper grades of elementary school shared toothpaste with their parents, with the proportion increasing with age [13]. In the present study as well, the proportion of non-fluoride toothpaste users increased the higher the grade level at school. In addition, this significance of grade level disappeared on adding the variables “anti-gingivitis”, “halitosis prevention” or “tartar control” alone (data not shown). Moreover, parents whose children were in grades 4-6 were more likely (*p* < 0.01, Chi-square test) to answer “anti-gingivitis”, “halitosis prevention” or “tartar control” as a reason for choice of toothpaste (data not shown). These results suggest the possibility of shared use of non-fluoride toothpaste between parents and children. The question of whether the child uses the same toothpaste as parents or not should be included in future studies [14].

For effective caries prevention by increasing the proportion of children using fluoride toothpaste, there is a pressing need first and foremost to disseminate correct information to parents and to educate them regarding the effectiveness of fluoride toothpaste, in order to encourage them to use it [15]. In the present study, no significant relationship was found between fluoride in the toothpaste being used and care taken for prevention of caries in children, so it appears unlikely that non-fluoride toothpaste users invariably have limited interest in caries prevention for their children. Parents should be able to understand that it is more important with children to take steps to prevent caries than gum disease and gingivitis. Also, if children use toothpaste less frequently because of the taste of the toothpaste, fluoride toothpaste with fruit flavors popular among children should perhaps be recommended.

In addition, parents might have intentionally selected non-fluoride toothpaste because they specifically did not want fluoride in their toothpaste, rather than lack of knowledge of the fluoride content of their toothpaste. There are people, including members of the Japan Federation of Bar Association and even some dental professionals, who are against using fluoride for caries prevention in Japan. Information regarding the side effects of fluoride is available in some magazines and websites. Some parents seem to dislike fluoride use and inevitably object if a school-based fluoride mouthwash program is introduced. An additional study is needed to clarify this issue.

The present study has a number of limitations. First, about 20% of parents did not respond and this may have led to bias. However, there is likely no selectivity in sex and age (Table 1). Second, nearly 15% of the questionnaires could not be used because one or more items were missing, and this biased the results. To address this concern, sensitivity analysis was conducted after adding “missing” categories. There was little change in the odds ratios and no change in statistical significance for the fully adjusted multilevel logistic regression model.

Third, only students from schools that agreed to cooperate were included in the study; it is possible that these schools take a more proactive stance toward dental health, so parents may have better dental knowledge and habits. Because the selection of schools may have been biased in the present study, it may not be possible to extrapolate our findings to all parents of Japanese schoolchildren. However, the percentage of fluoride toothpaste users among schoolchildren (6-14 years old) of the nationally representative sample in National Health and Nutrition Survey, Japan (86.3%) was close to that estimated in our study population (6-12 years old) (89.1%, 95% confidence interval: 88.6-89.7%) [5]. Moreover, there was no statistically significant variation among schools in the present study (data not shown: *p* > 0.05, a chi-squared test), and school- level variance (SE) was 0 (0) in the fully adjusted multilevel logistic regression model. Therefore, variation of the ratio of children using non-fluoride toothpaste in schools is negligible after adjustment for individual factors.

As there is no data regarding the period of toothpaste use, it is possible that the toothpaste being used at the time of the survey period simply happened to be either fluoride or non-fluoride. Finally, no information was obtained regarding the person who answered the questionnaire.

## Conclusions

Non-fluoride toothpaste use was significantly related to giving ‘anti-gingivitis’ as the reason for choice of toothpaste, and to not giving ‘has fluoride’, ‘is cheaper’ and ‘tastes good’ as reasons for choice of toothpaste.

## Competing interests

The authors declare that they have no competing interests.

## Authors’ contributions

JO and TY drafted the study, participated in its design, performed the statistical analysis and drafted the manuscript as principal authors. YA, JA, YH and SA participated in designing the study, helped with the data analysis and critically revised the manuscript. All authors read and approved the final manuscript.

## Pre-publication history

The pre-publication history for this paper can be accessed here:

http://www.biomedcentral.com/1472-6831/13/74/prepub
